# Caregiver strategies before intervention moderate caregiver fidelity and maintenance in RCT of JASPER intervention with autistic toddlers

**DOI:** 10.1002/jcv2.12247

**Published:** 2024-05-10

**Authors:** Wendy Shih, Amanda Gulsrud, Connie Kasari

**Affiliations:** ^1^ University of California‐Los Angeles Los Angeles California USA

**Keywords:** autism, caregiver, early intervention

## Abstract

**Background:**

Interventions facilitated by caregivers have gained popularity among those caring for young children with autism. Instructing caregivers on specific techniques to foster social communication skills in their at‐risk or diagnosed autistic children has the potential to alleviate concerns about their children's development. Moreover, it can offer a more intensive early intervention compared to what community providers alone can deliver. This study seeks to explore the correlation between caregiver strategies employed prior to participating in a caregiver‐mediated intervention and the caregiver's fidelity to the intervention, as well as its sustainability during the follow‐up period and child outcomes. This study constitutes a secondary analysis of a randomized controlled trial that compared the joint attention, symbolic play, engagement, and regulation (JASPER) and Psychoeducational Education Intervention (PEI), revealing significant advancements in children's social communication skills with the JASPER intervention.

**Methods:**

Eighty‐six children (average age 31.5 months) with ASD and their primary caregivers enrolled in the two armed randomized trial evaluating the effect of JASPER versus PEI. Generalized linear mixed models were used to model the longitudinal trajectories of the outcomes.

**Results:**

Results indicated that caregivers in the JASPER intervention made more gains in overall JASPER strategies and individual domain strategies (environment, prompt, communication, mirrored pacing) compared to the caregivers in PEI (*p*'s < 0.01) from baseline to exit. While both groups regressed some in overall and subdomain strategies at follow‐up, caregivers in the JASPER intervention maintained more overall, and specifically in communication, and mirrored pacing strategies compared to PEI group (*p*'s < 0.05). Further, baseline caregiver strategies moderated the treatment effect of child's joint attention skills from exit to follow‐up (*p* = 0.002), where JASPER dyads with high caregiver strategy use at baseline continued to improve in JA skills post exit, whereas all other children did not.

**Conclusion:**

In summary, understanding caregiver style of interaction before intervention on caregiver fidelity and maintenance from exit to follow up and child progress is important to improving intervention uptake and child outcomes.

## Graphical abstract

Interventions facilitated by caregivers have gained popularity among those caring for young children with autism. Instructing caregivers on specific techniques to foster social communication skills in their at‐risk or diagnosed autistic children has the potential to alleviate concerns about their children's development. Understanding caregiver style of interaction before intervention on caregiver fidelity and maintenance from exit to follow up and child progress is important to improving intervention uptake and child outcomes.

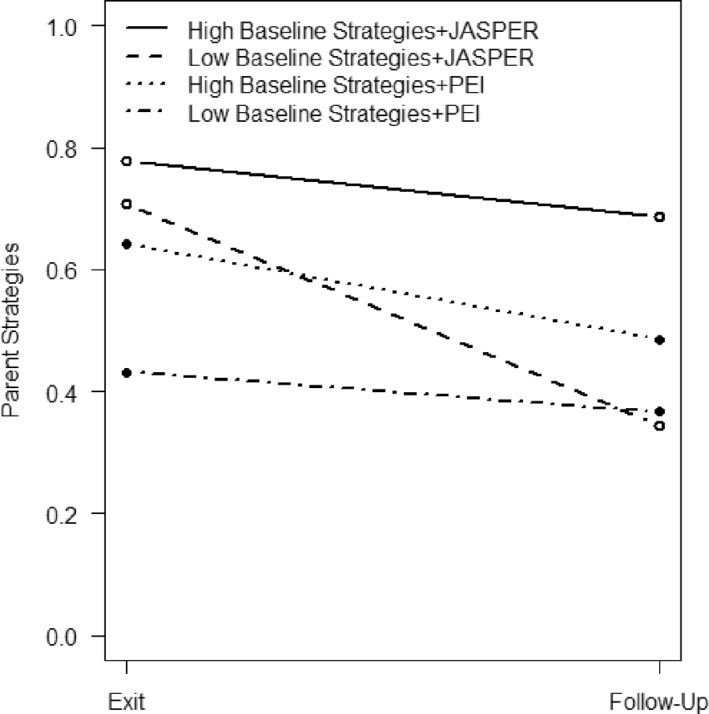

**Key points**

- Teaching caregivers specific strategies to help them teach their young children who are at risk or diagnosed with autism has the potential to offset some of the worry caregivers have about their children's development as well as providing a higher dose of intervention than current practices can accommodate with community therapists.
- The children of joint attention, symbolic play, engagement, and regulation (JASPER) caregivers who started with higher baseline strategies continued to improve in joint attention skills post intervention to follow‐up.
- Aligning the fit between the caregiver and the early intervention model matters.
- Caregivers with more initial strategies and received JASPER are able to maintain more strategies and their children have better joint attention skills after intervention ends.

## INTRODUCTION

Caregiver mediated interventions have grown in popularity for young autistic children and their families. Teaching caregivers specific strategies to encourage social communication skills in their young children who are at risk or diagnosed with autism has the potential to offset some of the worry caregivers have about their children's development, as well as provide a higher dose of early intervention than community providers alone can provide. Most caregiver mediated models applied to children with developmental disabilities fall within a naturalistic developmental, behavioral framework. These models combine responsive, developmental, and behavioral strategies, but vary in how much emphasis is placed on different domains and strategies. They also vary in how success of the intervention is measured; sometimes the success of intervention is based upon the caregiver response, the child response, or a combination of both. Despite our understanding that a single intervention is not effective for all dyads, few studies have focused on the active ingredients or mechanism for why a model may provide benefit, and for whom (caregivers and children) might benefit the most.

Although some effort has been made to examine common elements of caregiver‐mediated interventions, it may be the unique elements that drive the effects on social communication and language outcomes. Mechanistic studies (or why an intervention works) have rarely been reported. Of the two studies that have examined mechanism in parent implemented early interventions, both found specific aspects of parent behavior that drove the child outcomes (Gulsrud et al., 2016; Pickles et al., 2015). Of importance to the current study is the one reported by Gulsrud et al., 2016. Gulsrud found that of four categories of parent strategy use with toddlers receiving the joint attention, symbolic play, engagement, and regulation (JASPER) intervention, one had the greatest effect on changes in child‐initiated joint engagement, the primary intervention outcome. This category of mirrored pacing includes parent strategies of imitation, appropriate pacing, and expansion of the play‐based activities. It involves responding to the child contingently by selectively mirroring back the actions that prompted the social interaction. These strategies are core components of the JASPER approach, and the association between these strategies and joint engagement helps to inform how this intervention is driving change in children's social communication skills.

When teaching parents strategies to increase joint engagement, parents vary in how well they uptake the strategies (fidelity), and whether they generalize these strategies, and maintain them over time, after the researchers have left (maintenance). If the intervention itself has benefit to children's outcomes, fidelity to the intervention model is often associated with better child outcomes (Shire et al., 2015). However, child moderators also matter. Studies typically find younger children and those at the higher end of the developmental scale (more developed language or cognitive skills) prior to the intervention trial have better outcomes (Klinger et al., 2021; Rogers et al., 2012). Specific child characteristics are also associated with better social communication and language outcomes, including initiations of joint attention, object interest, play diversity and fine motor skills (Panganiban & Kasari, 2022; Yoder & Stone, 2006). These child characteristics are important to consider given our knowledge of the skill heterogeneity of autistic children, and that not all children respond to the same intervention in the same way.

In much the same way as children may respond differently to an intervention, caregivers also vary in how well an intervention model may fit their expectations, acceptance, and interaction style. Most models follow some predetermined schedule of topics in a week‐to‐week flow, but few models measure how closely the parent already demonstrates the strategies to be taught in the intervention model prior to beginning a research trial and whether beginning strategies consistent with the model makes a difference in better fidelity to model elements, effects on child outcomes, and sustainability of intervention strategies after the study. In one study, Shire et al. (2016) examined the relationship between caregiver interaction style (directiveness vs. responsivity) at baseline and child outcomes within caregiver‐child interactions over the course of a caregiver‐child intervention. They found that caregivers' interaction style was differentially associated to child gains in joint engagement. Caregivers who used strategies that were more responsive had children with greater gains in *child initiated* joint engagement; while, caregivers who were more directive had children with greater gains in *adult initiated* joint engagement. Hence, focusing on how parents naturally foster joint engagement with their children has critical implications especially for caregiver‐mediated interventions.

Therefore, the current study examines both caregiver and child behaviors prior to the start of a caregiver mediated intervention, and how those behaviors moderate treatment outcomes at intervention end, and sustainability of strategy use during the follow up period. We also examine the effect of the parents own feelings of comfort and ease of implementation as it relates to their adoption of the strategies during the intervention trial and maintenance after.

### Study design

**FIGURE 1:**
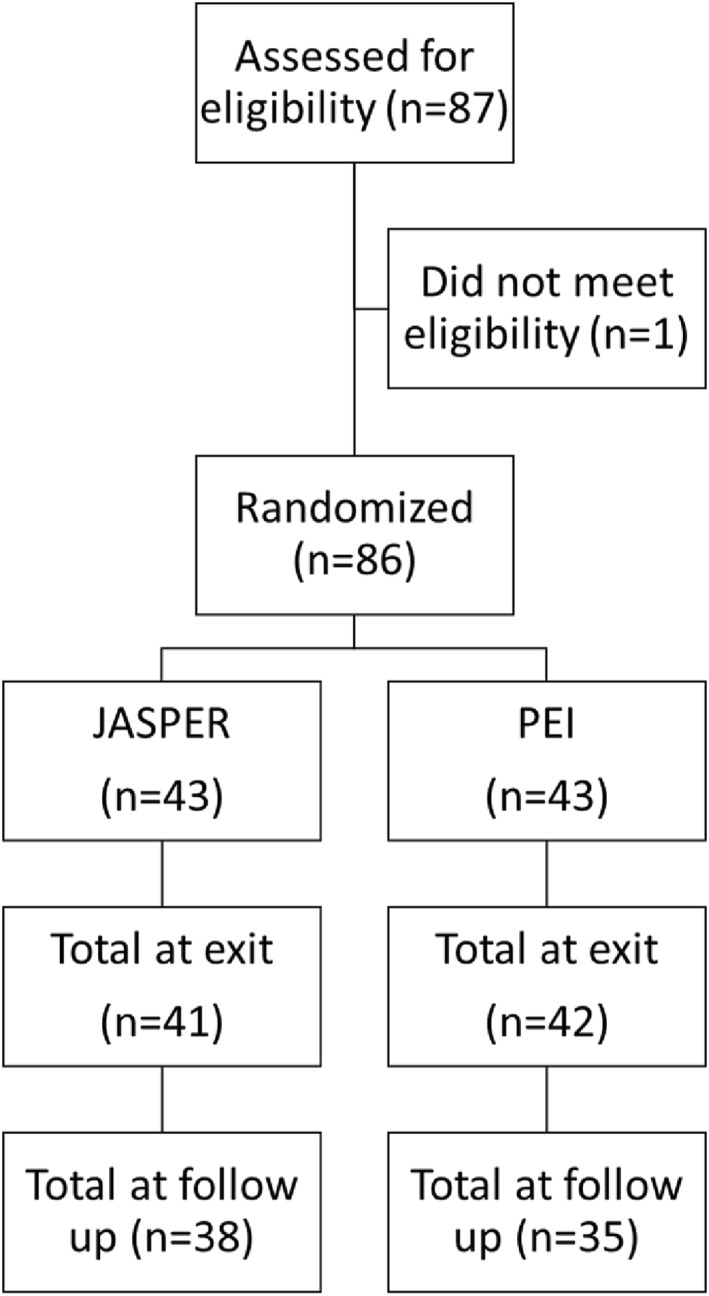
Original RCT consort chart.

The current study is a secondary data analysis from a two‐arm randomized controlled single site trial where children were randomized to one of two treatment conditions, caregiver mediated JASPER, or psychoeducational intervention based on Brereton & Tonge early intervention for parents (psychoeducational education intervention [PEI]) (Kasari et al., 2015; See Figure 1) in a clinic setting with a 1‐1 ratio from 2007 to 2012. Randomization was conducted by an independent data‐coordinating center. All children were also involved in the same ABA‐based early intervention program of 30 h per week (Discrete Trial Training and other ABA interventions in the context of a classroom). The sample size (*n* = 86) was powered for the original RCT to evaluate JASPER as superior to caregiver education on social communication and language outcomes in children (Kasari et al., 2015; See Figure 1).

The JASPER intervention included the primary caregiver and his/her child for 1 hour per week for a total of 10 weeks (two sessions of 30 min per week) with active hands on coaching of the caregiver by a trained interventionist. JASPER is a manualized developmental and behavioral intervention involving active coaching of caregivers to use strategies for setting up the learning environment, modeling and prompting for joint attention, expanding play, and using developmentally appropriate language (Kasari et al., 2021).

The psychoeducation (PEI) intervention provided 1:1 interventionist meetings with the caregivers in informational sessions of 1 hour per week for 10 weeks without the child being present (Brereton & Tonge, 2005). Sessions covered specific topics each week and caregivers were able to ask questions specific to their own child's development directly with their therapist. The content of the manualized intervention included several topics such as information on autism, details of specific strategies for behavioral challenges, principles of managing behavior, strategies for teaching new skills, improving social interaction and communication, managing caregivers' stress.

### Measures

*Caregiver–child interaction*. A 10‐min videotaped interaction was collected for each caregiver–child dyad prior to the start of intervention, at the end of intervention (10 weeks later) and 3 months post intervention. Caregivers were asked to engage in free play with their child as they normally would at home using a standard set of toys (including dolls, dishes, puzzles, trucks, shape sorter, blocks). These materials were separate from the materials used in intervention sessions. The videotapes were coded by coders blinded to group status. Videos were coded for caregiver use of strategies and periods of joint engagement adapted from Adamson et al. (2004). Reliability was established among independent coders for total time in joint engagement (i.e. jointly engaged based on codes of supported and coordinated joint engagement) on a random 25% of videotapes across time points, conditions, and participants (intraclass correlation: ICC = 0.95).

Caregivers' strategy use was also rated during the 10‐min caregiver child interaction by blinded, independent coders. The rating was developed to reflect the caregivers' fidelity to strategies taught in the JASPER training protocol and are also common and core strategies in many Naturalistic Developmental Behavioral Interventions (e.g. NDBI‐Fi, Frost et al., 2020 and the MONSI‐CC, Vibert et al., 2020). Four composite codes were included in the analyses: environmental arrangement, mirrored pacing, prompting, and communication. Each of the behaviors was rated for presence or absence in each of the 2‐min intervals dividing by the total possible behaviors in each category, yielding a proportion *e* score between 0 and 1 where score of 1 indicate good demonstration of the strategy (ICC = 0.92; Gulsrud et al., 2016). Environment category included behaviors such as “did the parent minimize overt distractions” or “was the caregiver at the child's eye level”. Mirrored pacing included behaviors such as “were the child's play acts imitated” or “did the caregiver immediately follow child's play acts”. Prompting included behaviors such as “did the parent prompt when needed” or “were the prompts appropriately timed/paced”. Communication category included “did the parent imitate or expand the child's language” or “were non‐directive” (See Fuller, 2013; Gulsrud et al., 2016). Overall total scoress were also computed that encompass all the individual behaviors within each category (i.e. environment, mirror pacing, prompting, communication) and overall caregiver strategy use.

The child's play behaviors recorded during the caregiver‐child interaction were coded for types of simple, combination, pre‐symbolic, and symbolic play acts (Lifter et al., 1993). Each occurrence of a play type was coded for number of occurrences in frequency counts. The total number of types (representing diversity of play) was summed to create a total frequency count (i.e. play diversity).

Child's frequency of initiating joint attention (IJA) skills was also coded in the caregiver‐child interaction (e.g., coordinated joint looks, pointing to share attention, giving, and showing). The frequency of s*pontaneously initiated* joint attention skills was collapsed into a summary variable of IJA. Independent coders, blinded to treatment condition, coded the videotapes according to a protocol used in previous studies (Harris et al., 1996; Kasari et al., 2006, 2008; ICCs' >0.95).

*Caregiver Diary*. The caregiver diary (Kasari et al., 2010) asks the caregiver to answer the extent to which they are using the strategies they are learning each week. Four questions address caregiver adherence and two questions address caregiver competence. Each question is rated on a 1 to 5 Likert scale where high scores indicate greater difficulty in implementing the learned strategies. This study utilizes the diaries collected post intervention. The overall summary score was *reversed scored* and averaged across the six items such that higher scores indicate greater confidence with implementing the learned strategies (Cronbach's *α* = 0.82).

*The Mullen Scales of Early Learning* (*MSEL*, Mullen, 1995). The MSEL was used to assess general cognitive ability for each child. The MSEL yields an early learning composite score based on scores for visual reception, gross motor, fine motor, and receptive and expressive language.

### Participants

Eighty‐six children between the ages of 22–36 months participated, with a clinical diagnosis of autism confirmed by independent testers with the ADI‐R (Lord et al., 1993) and ADOS (Lord et al., 2000), and no significant physical disabilities or uncontrolled seizures. On average, these children were 31.5 months, primarily male (81%), over half Caucasian (63%), and were recruited from the same early intervention program consisting of 30 h per week of combined behavioral, speech and occupational therapies. Caregivers included 76 mothers, 8 fathers, and 1 grandmother. Over 76% of the caregivers were college graduates or received graduate/professional training.

### Statistical approach

Generalized linear mixed models (GLMM) with main effects of treatment (JASPER and PEI) and time (baseline, end of treatment, and follow‐up), treatment by time interactions and subject level random intercepts were used to model the longitudinal trajectories of the outcomes, employing an identity link for continuous outcome variables and a log link function for count outcomes (using SAS software MIXED and GLIMMIX procedures respectively). Time was modeled such that the rate of improvement (slope) over the treatment phase (baseline to end of treatment) was allowed to differ from that over the follow‐up phase (end of treatment to the 3‐month follow‐up, broken line model). GLMM accounts for correlations between repeated measures within subjects, easily allow for both fixed and time‐varying covariates and automatically handle missing data, producing unbiased estimates as long as observations are missing at random. Hence, all available observations from each subject were utilized in modeling via the GLMM. The associations between caregiver strategies with child longitudinal outcomes were also examined using GLMM. Moderation analysis extended the GLMM models by including a 3‐way interaction (treatment × time × baseline caregiver strategies), all its lower term interactions, and main effects. The moderation analysis utilized all participants' data. Effect sizes (ES) are reported using Cohen's *f* in the results section where ES of 0.10, 0.25 and 0.40 are generally regarded as small, moderate, and large (Cohen, 1992).

## RESULTS

Baseline treatment differences on child and caregiver characteristics were evaluated and only children's age was found to be statistically different between the PEI and the JASPER group. Children in the PEI group were on average older by 1.6 months (See Table 1).

**TABLE 1:** Child and caregiver characteristics.

Child and caregivers characteristics	Total (*n* = 86)	PEI (*n* = 43)	JASPER (*n* = 43)	*p*‐value
Child's age (month): Mean (SD)	31.49 (3.19)	32.33 (2.63)	30.65 (3.50)	0.025
Child's gender: *n* (%)				0.999
Female	16 (19%)	8 (19%)	8 (19%)	
Male	70 (81%)	35 (81%)	35 (81%)	
Child's race/ethnicity: *n* (%)				0.448
African American	2 (2%)	2 (5%)	0 (0%)	
Caucasian	53 (61%)	26 (60%)	27 (63%)	
Hispanic	7 (8%)	4 (9%)	3 (7%)	
Asian/Pacific Islander	10 (12%)	6 (14%)	4 (9%)	
Other/mixed	14 (17%)	5 (12%)	9 (21%)	
Mullen scales of early learning: Mean (SD)				
Visual receptive age equivalency	24.13 (9.13)	25.37 (9.28)	22.88 (8.91)	0.467
Fine motor age equivalency	22.48 (5.09)	22.88 (5.32)	22.07 (4.87)	0.524
Receptive language age equivalency	18.10 (10.97)	19.21 (11.53)	17.00 (10.39)	0.416
Expressive language age equivalency	17.19 (9.44)	18.21 (9.46)	16.16 (9.41)	0.295
Early learning composite	68.05 (20.32)	68.09 (20.60)	68 (20.28)	0.890
Mother's age (years): Mean (SD)	35.83 (4.60)	34.17 (4.56)	36.88 (4.45)	0.076
Mother's education: *n* (%)				0.200
High school	3 (3.49%)	3 (6.98%)	0 (0%)	
Some college	14 (16.28%)	8 (18.60%)	6 (13.95%)	
Special training after high school	2 (2.33%)	2 (4.65%)	0 (0%)	
College	38 (44.19%)	17 (39.53%)	21 (48.84%)	
Graduate/professional training	28 (32.56%)	12 (27.91%)	16 (37.21%)	
Do not wish to disclose	1 (1.16%)	1 (2.33%)	0 (0%)	

### Is there a treatment difference in caregivers' use of JASPER strategies from baseline to end of intervention and follow‐up?

**FIGURE 2:**
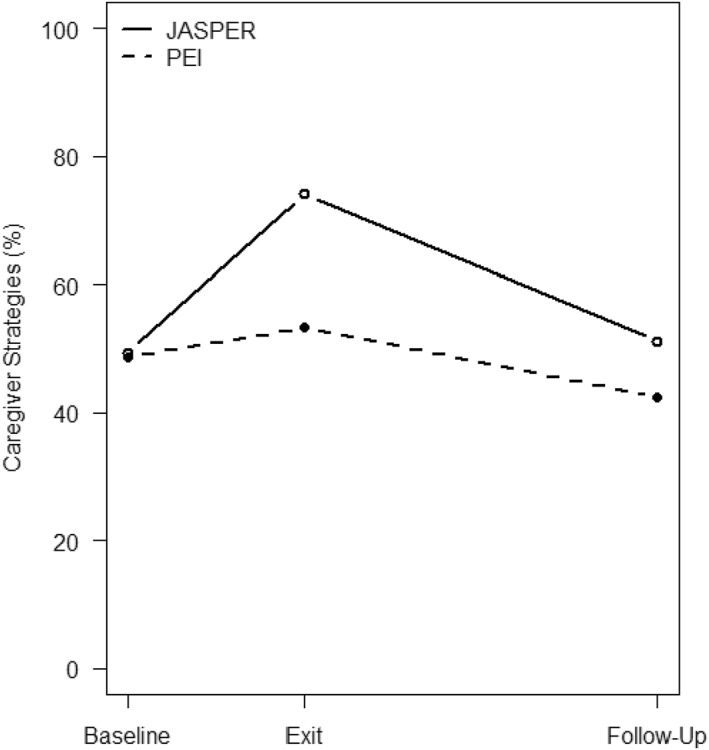
Treatment evaluation of JASPER caregivers' strategies.

Total Caregiver Strategy: On average, caregivers use 49% of the JASPER strategies at baseline with no significant difference between the two treatment groups. From baseline to the end of intervention, there is a significant treatment effect (F(1,151) = 36.45, *p* < 0.001, ES = 0.49) on caregivers strategy use where JASPER caregivers made more gains in JASPER strategies compared to PEI caregivers (See Table 2). While both groups used fewer strategies at follow‐up, JASPER caregivers still maintained more strategies at follow‐up (slightly but significantly greater than baseline) (F(1,151) = 2.83, *p* = 0.0053, ES = 0.14; see Figure 2).

**TABLE 2:** Caregiver strategies by treatment group.

	PEI	JASPER
Overall caregiver strategies		
Baseline	48.6% (10.8%)	49.5% (11.1%)
Exit	53.1% (13.5%)	74.2% (16.4%)
Follow‐up	42.2% (8.4%)	51.2% (15.7%)
Environment strategies		
Baseline	69.7% (13.5%)	70.4% (14.4%)
Exit	72.7% (12.7%)	88.8% (10.6%)
Follow‐up	68.5% (12.2%)	73.4% (15.5%)
Prompt strategies		
Baseline	47% (12.7%)	47.1% (14.3%)
Exit	49.9% (17.3%)	68.5% (23.9%)
Follow‐up	35.7% (5.4%)	40% (13.2%)
Communication strategies		
Baseline	47.1% (13.7%)	48.7% (16.8%)
Exit	50.9% (16.3%)	72.2% (23.4%)
Follow‐up	34.9% (11.3%)	43.2% (20.7%)
Mirrored pacing		
Baseline	30.7% (27.8%)	31.8% (22.6%)
Exit	38.8% (29.7%)	67.4% (25.5%)
Follow‐up	29.8% (21.4%)	48.2% (31.4%)

**FIGURE 3:**
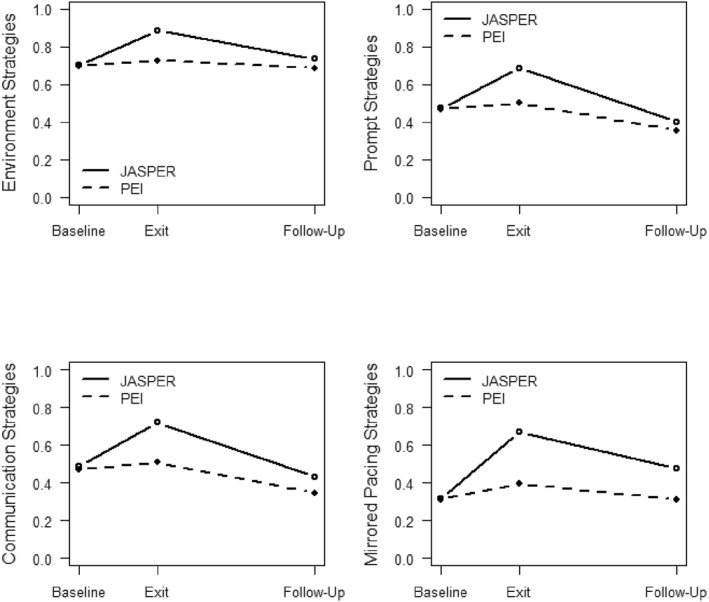
Treatment differences by individual subdomain strategies.

Subdomain Strategies: Environment, Prompting, Communication, and Mirrored Pacing: Similarly, there were significant treatment effects on all 4 subdomain strategies where JASPER caregivers made significant gains in all subdomain JASPER strategies whereas thePEI caregivers remained stable (environment: F(1,151) = 35.94, *p* < 0.001, ES = 0.49; prompting: F(1,151) = 56.37, *p* < 0.001, ES = 0.61; communication: F(1,151) = 18.69, *p* < 0.001, ES = 0.35; mirrored pacing: F(1,151) = 15.47, *p* < 0.001, ES = 0.32) from baseline to end of intervention. At follow‐up, the JASPER caregivers demonstrated fewer environment and prompt strategies and were no longer significantly different compared to PEI caregivers (F(1,151) = 2.94, *p* = 0.117, ES = 0.14; F(1,151) = 1.40, *p* = 0.239, ES = 0.096; See Figure 2). In addition, both groups also decreased strategy use at follow‐up regarding their communication and mirrored pacing strategies. However, JASPER caregivers still maintain more communication and mirrored pacing strategies at follow‐up compared to the PEI caregivers (F(1,151) = 4.32, *p* = 0.039, ES = 0.17; F(1,151) = 6.97, *p* = 0.009, ES = 0.21; see Table 2 and Figure 3).

### Do baseline caregiver strategies *moderate* treatment difference on the sustainability of strategy use from end of intervention to follow‐up?

**FIGURE 4:**
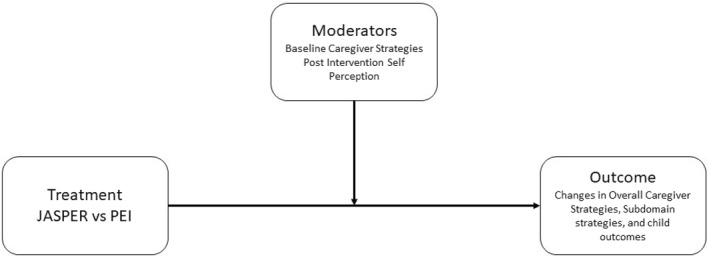
Moderation design Figure.

*Baseline Total Caregiver Strategies*: Baseline caregiver strategy use moderated the maintenance of treatment effect from exit to follow‐up (F(1,68) = 4.70, *p* = 0.034, ES = 0.26, see Figures 3 and 4). Among caregivers with higher baseline JASPER caregiver strategies, caregivers randomized to JASPER had significantly higher caregiver strategy scores at exit compared to PEI caregivers (F(1,68) = 9.23, *p* = 0.003, ES = 0.37). Similarly, among caregivers with low JASPER baseline caregiver strategy use, caregivers randomized to JASPER had significantly higher caregiver strategy scores at exit compared to PEI caregivers (F(1,68) = 11.66, *p* = 0.001, ES = 0.42).

**FIGURE 5:**
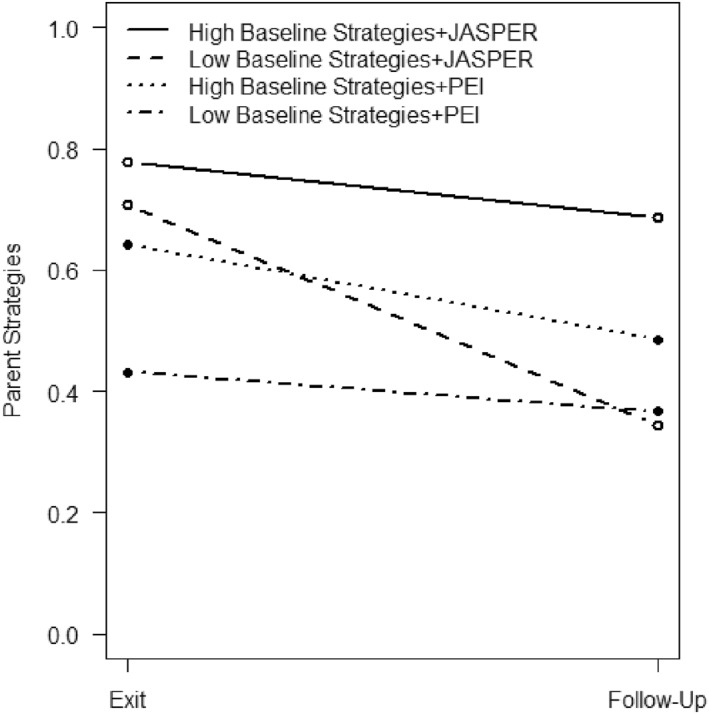
Baseline strategies moderating maintenance of treatment effect on caregiver strategies from post intervention to follow‐up.

Within the JASPER caregiver group, there were no significant differences in overall caregiver strategy use (e.g., fidelity) at exit regardless of whether they started with higher or lower strategies at baseline (F(1,68) = 0.55, *p* = 0.459, ES = 0.09). Yet, JASPER caregivers who started with high strategies at baseline were able to maintain their strategy use more at follow‐up compared to JASPER caregivers who had lower strategy use at baseline (F(1,68) = 11.82 *p* = 0.001, ES = 0.42; see Figure 5).

Further, within PEI caregivers, PEI caregivers who started with higher JASPER strategy use at baseline had higher JASPER strategy use at exit compared to PEI caregivers who had lower strategy use at baseline (F(1,68) = 4.48, *p* = 0.036, ES = 0.26). However, there were no significant differences in overall strategy use at follow‐up between PEI caregivers who started with high or low strategies at baseline (F(1,68) = 1.06, *p* = 0.306, ES = 0.12).

*Baseline caregiver strategies moderate treatment difference on subdomain strategies from end of intervention to follow‐up*: Moderation of baseline total caregiver strategies with treatment effect on individual subdomain strategies from *end of intervention to follow‐up* was also examined. Baseline overall caregiver strategies moderated treatment effect from end of intervention to follow‐up (F(1,68) = 7.75, *p* = 0.007, ES = 0.34) for mirrored pacing strategies only and not for the other individual subdomain strategies (i.e. environment, prompting, and communication). Among JASPER caregivers, there was no difference in mirrored pacing strategies at exit between caregivers with high or low strategies at baseline (F(1,68) = 0.410, *p* = 0.527, ES = 0.08), but there was a significant difference in mirrored pacing strategies at follow‐up, where JASPER caregivers with high baseline strategies maintained higher mirrored pacing strategies at follow‐up compared to JASPER caregivers with low baseline strategies (F(1,68) = 7.51, *p* = 0.008, ES = 0.33).

**FIGURE 6:**
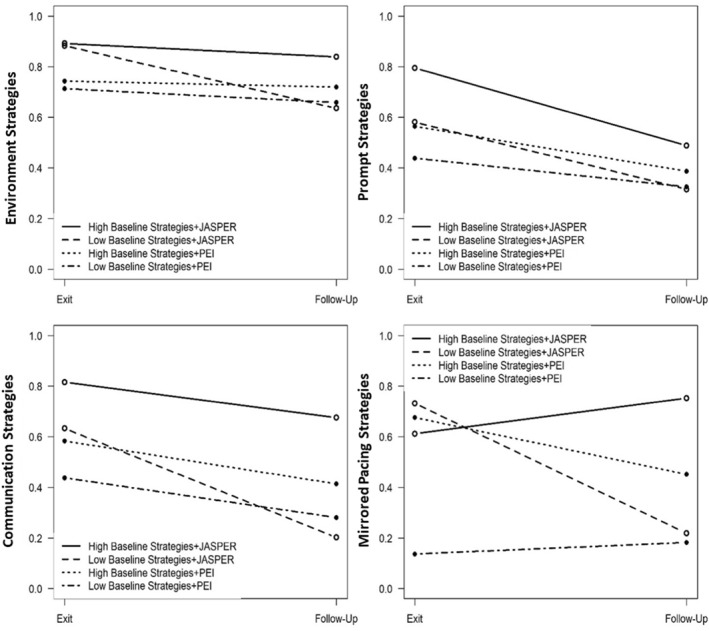
Baseline strategies moderating maintenance of treatment effect post exit on individual strategies.

On the other hand, PEI caregivers with higher JASPER strategies at baseline had significantly higher mirrored pacing strategies at exit compared to PEI caregivers with lower JASPER strategies at baseline (F(1,68) = 7.90, *p* = 0.007, ES = 0.34; see Figure 5). This difference did not maintain at follow‐up as there was no significant difference in mirrored pacing strategies at follow‐up between PEI caregivers with high or low baseline JASPER strategies (F(1,68) = 1.44, *p* = 0.234, ES = 0.15) (Figure 6).

### After the trial ends: Do *baseline* caregiver strategies and *post intervention* self‐perception of implementation moderate treatment effects on child outcomes

*Baseline caregiver strategies as moderator*: Moderation of baseline caregiver strategies by treatment on child social communication outcomes (joint engagement, joint attention skills, and play diversity) was also examined. While baseline strategies did not moderate treatment effect on joint engagement and play diversity post intervention, baseline strategies did significantly moderate treatment effect on total joint attention skills from post intervention to follow‐up (F(1,67) = 11.02, *p* = 0.0015, ES = 0.41). The children of caregivers who started with high baseline JASPER strategies and were randomized to the JASPER intervention continued to improve significantly in IJA post intervention to follow‐up (F(1,67) = 7.20, *p* = 0.009, ES = 0.32); whereas, children of caregivers who started with high baseline JASPER strategies in PEI decreased significantly in IJA post intervention to follow‐up (F(1,67) = 19.09, *p* < 0.001, ES = 0.53). Children of caregivers who started with low baseline JASPER strategies remained stable in IJAs post intervention regardless of intervention received (JASPER: F(1,67) = 2.74, *p* = 0.103, ES = 0.20; PEI: F(1,67) = 0.34, *p* = 0.564, ES = 0.07).

*Post intervention self‐perception measured from caregiver diary as moderator*: Caregiver self‐perception of their implementations post intervention moderated treatment effect on IJA from post intervention to follow‐up (F(1,57) = 3.61, *p* = 0.062, ES = 0.25). JASPER caregivers who perceived themselves as struggling with implementing the strategies at home (i.e. low diary scores at post intervention) had children who remained stable in their joint attention skills from post intervention to follow‐up (F(1,57) = 19.09, *p* = 0.477, ES = 0.58). Whereas, the same PEI caregivers (i.e. low diary scores at post intervention) had children who decreased in their joint attention skills from post intervention to follow‐up (F(1,57) = 11.09, *p* = 0.002, ES = 0.44). In contrast, JASPER caregivers who reported more confidence in implementing the learned strategies had children who improved in their joint attention skills (F(1,57) = 27.29, *p* < 0.001, ES = 0.69). The same PEI caregivers (i.e. high diary scores) had children who remained stable post intervention (F(1,57) = 0.001, *p* = 0.964, ES = 0.004).

## DISCUSSION

The results of this study highlight how variability in caregivers' interaction strategies with their children before beginning an intervention trial matter in the progress made of both caregiver (fidelity and sustainment of strategies) and their child's progress in social communication skills. Such findings may reflect “fit or acceptance between the caregiver and the early intervention model”. Three findings were noted. First, regardless of how closely the caregiver's initial strategies matched fidelity of the intervention model, if caregivers were randomized to the intervention model (in this case JASPER) they ended with high levels of JASPER strategy use (i.e., fidelity to the intervention model). Thus, lower beginning levels of fidelity to the JASPER model did not hinder caregivers in obtaining high strategy adherence to the model at the end of the trial. Higher fidelity to JASPER strategies in the beginning for the comparison PEI group caregivers did not increase those strategies over the course of the trial suggesting that information alone does not translate to actual interactions with their children. It should be noted that none of the caregivers were already at high fidelity across all dimensions of the parent mediated model prior to randomization, and that fidelity to the model did result in greater child outcomes.

Regardless of fidelity to JASPER strategies at end of the active phase, caregivers also vary in how well they sustain the strategies they have learned. A second finding, then, is that those caregivers with higher initial fidelity to JASPER, suggesting a better fit between the model and their parent‐child interaction strategies, who also received the JASPER intervention maintained more of their strategies during the follow up phase. Comparison group caregivers and those randomized to JASPER with initial low fidelity sustained or demonstrated fewer of the strategies in the follow up phase. While initial fit may be important in later sustainability, it is noted that all caregivers decreased in their use of strategies during the non‐active, follow up phase. There may be several explanations for this decrease. One is that caregivers may just need more time with a consultant to solidify the strategies with 20–24 sessions too few overall especially when the strategies are new or just not employed prior to the intervention. However, it is also possible that the researcher needed to find ways to help the parent integrate the strategies better into the daily lives of their children.

Finally, JASPER randomized caregivers with high beginning levels of JASPER strategies and caregivers who found the strategies easy to implement had children who continued to improve in their joint attention skills from the end of the intervention to the follow‐up whereas children of caregivers with low beginning levels of JASPER strategies and those who struggled with implementing strategies at post intervention, regardless of intervention assignment, did not change in their joint attention skills from treatment end to follow‐up. Joint attention skills are important in language acquisition; thus, joint attention (and particularly spontaneous initiations of joint attention as measured here) becomes an important target for toddlers who were all language delayed as in this study (Shih et al., 2021).

Also of note, gains were greater for both groups of children during the active intervention phase, a common finding in brief intervention trials (Kasari et al., 2023). Continued application of intervention strategies are important so that children continue to make the progress at the rate of the active phase, but there can be several reasons why the intervention strategies are difficult to maintain. One issue for caregivers is that as their children continue to grow developmentally, the intervention targets also change. Brief parent mediated interventions are useful for jumpstarting better engagement and social communication development in their children, but professionals are often still needed to advise parents on appropriate developmental targets. Another reason may be that as children left the clinic‐based school setting, they transitioned to community intervention programs that may have varied in quality, intensity, and approach. It is sometimes harder to maintain intervention strategies that may be at odds with the delivery of community interventions. Parents may receive differing advice on approach and content of intervention once they transition to school or community‐based intervention programs.

## CONCLUSION

This study highlights that how caregiver's interact with their autistic children prior to beginning an early intervention research trial is associated with how well they uptake and continue the intervention (fidelity and sustainment). Fidelity and sustainment of an evidence ‐based intervention, such as JASPER, is also associated with child social communication outcomes. While caregivers can reach fidelity during the active phase of intervention, these initial caregiver strategies are also associated with sustainment and continued child progress during the follow up phase. Future studies will want to consider ways in which an intervention may need to be adapted or intensified in order to help all caregivers sustain strategies that may help their children's development. It will also be important to follow children into their next settings and measure the quantity, quality and approach of community interventions and children's responses to maximize development.

## AUTHOR CONTRIBUTIONS

**Wendy Shih**: Formal analysis; validation; visualization; writing – original draft; writing – review & editing. **Amanda Gulsrud**: Conceptualization; data curation; supervision; writing – original draft; writing – review & editing. **Connie Kasari**: Conceptualization; data curation; funding acquisition; investigation; project administration; supervision; writing – original draft; writing – review & editing.

## CONFLICT OF INTEREST STATEMENT

Connie Kasari and Amanda Gulsrud receive royalties for the JASPER manual.

## ETHICAL CONSIDERATIONS

All study procedures were approved by the Institutional Review Board (IRB) at the University of California—Los Angeles (IRB#11‐000032). Written consent from all enrolled participants was obtained. The study protocol was pre‐registered in ClinicalTrials.gov (https://classic.clinicaltrials.gov/ct2/show/NCT00999778).

## Data Availability

Please email author—Connie Kasari for any data requests.
